# 

**Published:** 1884-07

**Authors:** 


### EDITORIAL.

### American Dental Association.

The twenty-fourth annual meeting of the American Dental Association will be held at Saratoga Springs, N. Y., commencing Tuesday, August 5th, at 10 A. M.

Geo. H. Cushing, Recording Sec'y.

### National Association of Dental Examiners.

The second annual meeting of the National Association of Dental Examiners will be held at Saratoga Springs, N. Y., on Friday evening, August 8, 1884.

All Boards of Examiners are urgently requested to send representatives to this meeting, as matters of great importance are expected to be brought before it.

GEO. H. CUSHING, Secretary.

### Pennsylvania State Dental Society.

The sixteenth annual meeting of the Pennsylvania State Dental Society will be held at Wilkesbarre, Pa., July 29th, 1884, and continue three days. Special rates at the Wyoming Valley Hotel; excursion rates over all the principal railroads in the State. Orders for the same can be had by addressing

W. H. FUNDENBERG, Corresponding Secretary.
958 Penn. Ave., Pittsburg, Pa.

### Dentists Benevolent Association.

The first annual meeting of the DENTISTS BENEVOLENT ASSOCIATION will be held at Sweet Springs, Saline Co., Missouri, Thursday, July 10th, 1884.

This association is composed exclusively of dentists and dealers in dental goods, and has for its object financial aid and relief to the families of deceased members. Every member of the profession should be interested in such a movement, and is cordially invited to be present and lend a helping hand toward future success.

As the meeting takes place during the session of the Missouri State Dental Society arrangements have been made for reduced railroad and hotel rates. Any one desiring to attend are requested to communicate with the secretary.

R. I. PEARSON, Secretary, C. H. DARBY, President,
2206 Troost Ave, Kansas City, Mo. St. Joseph, Mo.

### Delegates to the American Dental Association.

The following Delegates to the American Dental Association have been appointed to attend the next meeting at Saratoga, first Tuesday in August, 1884, by the Executive Committee of the Mississippi Valley Dental Association:

Wm. D. Kempton, C. M. Wright, N. S. Hoff, J. W. Jay, D. Gale French, F. W. Sage, R. J. Corson, C. I. Keely, O. N. Heise, H. L. Moore, Samuel Wardle, F. T. Emminger, Wm. Van Antwerp, Jas. G. Reid, E. J. Waye, J. G. Cameron, J. S. Cassidy, J. F. Canine, A. G. Rose, M. H. Fletcher, J. U. Paine, J. G. Templeton, E. G. Betty.

### Society Meetings.

### MINNESOTA STATE DENTAL SOCIETY.

The first annual meeting of the State Dental Society of Minnesota will be held at St. Paul, commencing Wednesday, July 16th, at 10 o'clock, A. M., and continue three days. An interesting programme has been prepared, which contains the following subjects, upon which there will be papers and discussions:

1st. Assimilation.
2d. Diseased Gums and Treatment.
3d. Gold Fillings.
4th. Articulation.
5th. Continuous Gum.
6th. Regulating.
7th. Artificial Crowns.

There will be demonstrations of continuous gum work by J. L. Jacobs, and mounting artificial crowns by W. N. Murray.

The profession in the State of Minnesota is starting in, in earnest to establish a good dental society, and doubtless success will attend the effort, for there are in that State as good dentists, scientifically and practically, as are to be found anywhere.

Members of the profession from other States are cordially invited.

St. Paul, with its surroundings, is well worth a visit, and should be a strong inducement to those outside to make a visit at this opportune time.

Let all be present who can, and give the weight of their presence and influence as a stimulus to make this meeting successful.

---

### PENNSYLVANIA STATE DENTAL SOCIETY.

The sixteenth annual meeting of the Pennsylvania State Dental Society will be held at Wilkesbarre, Pa., commencing July 29th, at 10 o'clock A. M., and continue three days.

This is one of our old, well-established societies, and has done a great work for the profession of the State.

Essays upon the following subjects will be read:

1st. Classification of Dental Tissues, Enamel, and Dentine.

2d. Dental Diagnosis.

3d. Reflections on the Practical Bearing of the Germ Theories of Disease.

4th. Operative Dentistry.

5th. Our Failures.

The following subjects for discussion will be presented:

1st. Piorrhœa Alveolaris.

2d. Capping Exposed Pulps.

3d. Dental Prosthesis.

The examining board will meet those desiring license to practice, and to examine diplomas before registration.

Meetings of this society are always interesting and instructive. It usually has a good representation of the profession in the State; but, doubtless, the attendence might be larger than it has usually been, as is the case of every State society in the country.

As a rule, our dental societies are not appreciated and sustained as they ought to be. The profession in each State should have a pride in making the most possible of this instrumentality for its progress and improvement. No other agency can do the work that can be done by well organized State societies. Then let the profession of every State rally around its State society.

### CONTENTS.

### COMMUNICATIONS.

### THE DENTAL REGISTER,

A Monthly Magazine Devoted to the Interests of the

### DENTAL PROFESSION.

*Terms Reduced to $2.00 Per Year. Single Copies, 25 Cents.*

**EDITED BY PROF. J. TAFT, D.D.S.**

—AND—

**Published by SPENCER & CROCKER, Ohio Dental and Surgical Depot.**

The volume commences in January and closes in December.

The Register being issued monthly is always fresh, therefore Indispensable to the practitioner who desires to keep posted up with the new inventions and improvements which are daily developed. Neither expense nor effort will be spared to give value and variety to its contents. Articles will be illustrated with well executed engravings whenever they will add to the interest or assist to explanation.

Its extensive circulation and frequency of issue make it one of the BEST DENTAL ADVERTISING MEDIUMS in the United States.

All subscriptions must begin with January or July.

Local or special notices, five lines or less, will be inserted for $3 each insertion; over five lines, 50 cts. per line.

All advertising bills payable in advance, or at furthest, after the issue of the first number containing the advertisement. All transient advertisements to be paid for in advance.

☞CONTRIBUTIONS SOLICITED.

Exclusively Editorial Communications should be addressed to the Editor.

The latest number of the Register may be ordered with or without other goods at any time, and all letters should be addressed to

